# Analysis of the heterogeneity of the BCR H-CDR3 repertoire in the bone marrow and spleen of 3-, 12-, and 20-month old mice

**DOI:** 10.1186/s12979-021-00231-2

**Published:** 2021-04-12

**Authors:** Lina Ma, Xinxin Tao, Xiaoyan He, Peng Wang, Long Ma, Bin Shi, Xinsheng Yao

**Affiliations:** Department of Immunology, Center of ImmunoMolecular Engineering, Innovation & Practice Base for Graduate Students Education, Zunyi Medical University, Zunyi City, 563000 China

## Abstract

The number of central and peripheral B cells and their responsiveness are decreased in aged mice. The diversity of mice central and peripheral B cell repertoires with increasing age has not been elucidated. In this study, we demonstrated that there were significant differences in the usage of some *V*, *D*, and *J* genes in the BCR H-CDR3 repertoire of bone marrow B cells, spleen B cells and spleen memory B cells in 3-, 12-, and 20-month-old mice. In the productive, pseudogene, and out-of-frame sequences, bone marrow B cells had significant differences in 5′J trimming with age; peripheral spleen B cells and memory B cells had significant differences in N1 insertion, N2 insertion, P5’D insertion, and 5’D trimming with age. The BCR H-CDR3 repertoire diversity of mice bone marrow B cells, spleen B cells and spleen memory B cells decreased with increasing age. The proportion of overlap in bone marrow and spleen B cells, but not spleen memory B cells, of mice at different ages was lower at 3 months than at 12 and 20 months. This study is the first to report the homogeneity and heterogeneity of the CDR3 repertoire of central and peripheral B cells change as mice age, to further investigation of the decline and response of B cell immunity in young/middle/old-aged mice.

**Supplementary Information:**

The online version contains supplementary material available at 10.1186/s12979-021-00231-2.

## Introduction

The percentage of B cells and antibody category conversion and reorganization decrease with age [1, 2]. The response B cells produced by aging animals under the same intensity of antigen stimulation are 1/10–1/50 that produced by adult animals [3]. B cell clone proliferation changes with age, and the genetic lineage of B cells also undergoes corresponding dynamic changes. In particular, the increase in memory B cell clones is closely related to the immune system status of elderly individuals [4–6].

The diversity of the naïve BCR H-CDR3 repertoire is derived from the rearrangement of the germline *V(D) J* gene in the bone marrow [7] (high-frequency mutations in the periphery that increase the diversity of the B cell repertoire [8]). A shift of the reading frame during the rearrangement process can result in an out-of-frame sequence, and rearrangement of the *V(D) J* pseudogene will not produce a functional sequence [9], although an out-of-frame and pseudogene rearrangement failure on one chromosome may be the start *V(D) J* on another chromosome to continue rearrangement. With the application of HTS to the CDR3 repertoire of T/B cells, the dynamic changes in the body’s immune system can be explored by comparing the effective rearrangement of functional genes (in frame) and the rearrangement of the out-of-frame and pseudogene sequences.

Human studies have shown that the lifespan of B cells in elderly individuals is increased, and the production in the bone marrow is reduced [10, 11]. B cell expansion clones increase with age [12, 13]. The repertoire is closely related to changes in age before and after immunization in mice [14]. At present, how the homogeneity and heterogeneity of the CDR3 repertoire of central and peripheral B cells change as mice age is not fully elucidated.

In elderly individuals, immunoglobulins IgM and IgD are reduced, and naïve B cells are transformed into memory B cells [12]. Plasma cells produce reduced IgG antibodies, which limit memory B cell diversity [15, 16]. As the mice age increases, how the corresponding changes in the peripheral memory B cell repertoire has not been elucidated.

In this study, we selected 3-, 12-, and 20-month-old mice and used HTS to detect the bone marrow B cell, spleen B cell, and spleen memory B cell BCR H-CDR3 repertoire. The homogeneity and heterogeneity of the productive, pseudogene, and out-of-frame sequences in the BCR H-CDR3 repertoire were compared and analyzed. The main components and characteristics of the central and peripheral BCR H-CDR3 repertoire of mice at different ages were further studied.

## Results

### Preparation of mice spleen samples and tissue HE staining

**Fig. 1:**
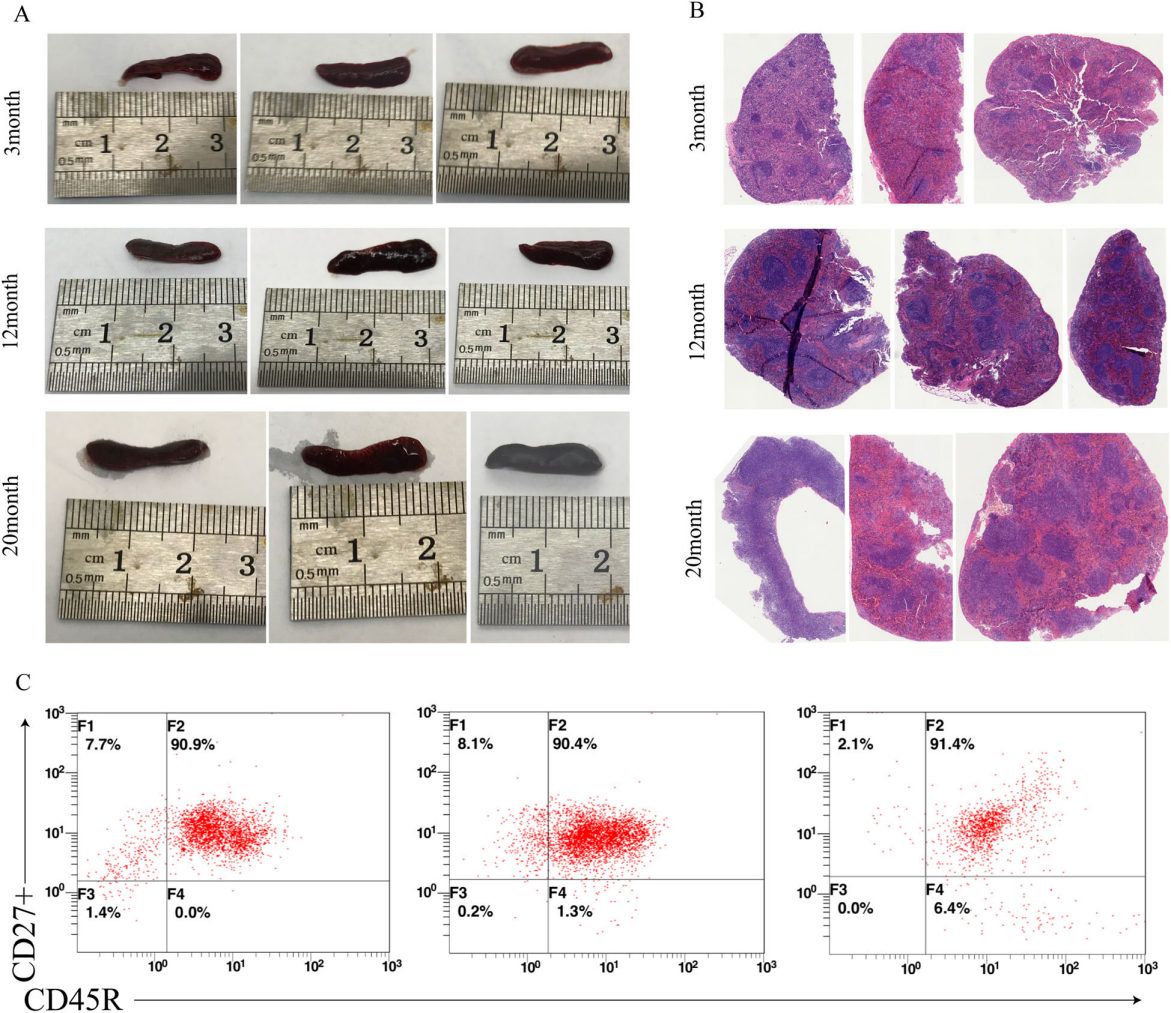
Preparation of mice samples of different ages. **a** Different ages of mice spleen. **b** HE stained sections of spleen tissues of mice at different ages (10X). **c** Spleen memory B cell sorting purity identification

The spleen tissue of mice aged 3, 12, and 20 months was taken (Fig. 1a): the spleen length was approximately 1.5–2 cm, and the color was bright red. The spleen white pulp structure became irregular with increasing age (Fig. 1b). The purity of spleen tissue memory B cells (CD45R^+^CD27^+^) by Miltenyi bead sorting was more than 85% (Fig. 1c).

### Mice bone marrow B cell, spleen B cell and memory B cell CDR3 repertoire

The concentration and purity of multiplex PCR products were in accordance with HTS requirements (Fig. S1). The CDR3 repertoire from the HTS of each tissue sample of 3-, 12-, and 20-month-old mice was compared and statistically analyzed according to the productive, pseudogene, and out-of-frame unique and total sequences (Table 1).

**Table 1:** The BCR H-CDR3 repertoire of productive, pseudogene, and out-of-frame sequences in mice of different ages

Sample	Productive Total (unique)	Pseudogene Total (unique)	Out of frame Total (unique)
M3BM1	3,662,734 (474483)	107,165 (20484)	350,156 (89830)
M3BM2	4,201,156 (522977)	128,828 (21769)	331,242 (87427)
M3BM3	772,633 (386000)	16,473 (16473)	137,409 (75974)
M3BM4	1,357,086 (177848)	50,529 (8549)	305,304 (54945)
M3BM5	1,296,498 (178251)	42,892 (8104)	348,632 (61586)
M12BM1	1,438,032 (197994)	35,419 (6960)	97,826 (30317)
M12BM2	1,477,622 (156699)	58,100 (8877)	231,830 (45326)
M12BM3	502,958 (64768)	16,480 (2915)	98,908 (20059)
M12BM4	1,108,553 (184165)	34,074 (7926)	207,506 (51285)
M12BM5	1,669,642 (170188)	66,465 (8764)	324,449 (50027)
M20BM1	1,494,519 (242290)	39,108 (9174)	400,643 (87675)
M20BM2	1,316,534 (199635)	45,487 (9149)	277,021 (58823)
M20BM3	561,490 (120788)	19,500 (5264)	120,708 (31740)
M20BM4	429,629 (76188)	11,326 (2883)	78,493 (17853)
M20BM5	313,694 (67759)	8452 (2542)	48,381 (13366)
M3S1	4,523,881 (786508)	149,238 (33361)	865,160 (208318)
M3S2	4,017,413 (682554)	108,054 (25438)	663,139 (213543)
M3S3	4,417,442 (637351)	137,097 (28256)	784,674 (160134)
M3S4	3,942,536 (606347)	97,789 (22957)	808,071 (164970)
M3S5	2,696,776 (571503)	63,029 (18062)	446,298 (122955)
M3S7	1,091,791 (91001)	44,851 (4866)	226,582 (27110)
M3S8	46,895 (12469)	1165 (446)	6682 (2261)
M3S9	183,534 (27923)	5709 (1241)	34,871 (6784)
M12S1	1,044,884 (101677)	15,109 (3242)	266,668 (20675)
M12S2	1,365,544 (280774)	42,482 (11209)	212,575 (60657)
M12S3	1,382,706 (226375)	28,890 (7327)	217,936 (51437)
M12S4	1,555,800 (265748)	23,832 (7127)	329,043 (70801)
M12S5	1,441,200 (201908)	24,061 (5619)	267,609 (51811)
M12S7	737,198 (137061)	44,046 (7571)	57,282 (21271)
M12S8	880,181 (169410)	14,891 (4214)	65,870 (23320)
M12S9	77,364 (11970)	1056 (330)	4592 (1626)
M20S1	1,296,983 (134233)	24,433 (4355)	564,603 (37880)
M20S2	1,646,868 (205593)	43,362 (8512)	235,117 (46844)
M20S3	535,873 (125903)	10,757 (3624)	100,049 (29217)
M20S4	1,000,811 (172421)	22,525 (5906)	246,581 (49540)
M20S5	579,776 (156417)	16,389 (5813)	116,676 (36376)
M20S7	197,807 (22819)	5327 (923)	114,176 (6685)
M20S8	717,148 (68896)	18,116 (2575)	634,418 (24260)
M20S9	55,512 (6239)	2232 (327)	11,120 (1928)

### Gene frequency in the BCR H-CDR3 repertoire

#### *V* gene usage (Fig. 2, Fig. S2–1 and Fig. S2–2)

(1) Productive sequences: 3-, 12-, and 20-month-old mice were given high-frequency *IGHV1–4*, *IGHV14–1*, *IGHV1–50*, and *IGHV1–64.* With the change in age, Bone marrow B cell *IGHV1–54* and *IGHV1–84*; spleen B cell *IGHV4–1*, *IGHV1–87*, *IGHV5–12*, and *IGHV9–1*; and spleen memory B cell *IGHV1–4*, *IGHV5–12–1*, and *IGHV5–9* showed significant differences at different months (*p* < 0.05). (2) Pseudogene sequences: 3-, 12-, and 20-month-old mice were given high-frequency *IGHV1–67* and *IGHV1–83*. Bone marrow B cell *IGHV1–25* and *IGHV1–83* and spleen B cell *IGHV1–32* and *IGHV1–67* showed significant differences with changes in age (*p* < 0.05). (3) Out-of-frame sequences: 3-, 12-, and 20-month-old mice were given high-frequency *IGHV1–4*, *IGHV14–1*, *IGHV1–50*, and *IGHV1–64*. Bone marrow B cell *IGHV11–1* and *IGHV14–1*; spleen B cell *IGHV11–1*, *IGHV1–4*, *IGHV1–87*, *IGHV1S-45*, and *IGHV9–1*; and spleen memory B cell *IGHV1–14*, *IGHV1–4*, *IGHV1–42, IGHV1–50*, *IGHV1–87*, and IGHV1S-45 showed significant differences with changes in age (*p* < 0.05).

**Fig. 2:**
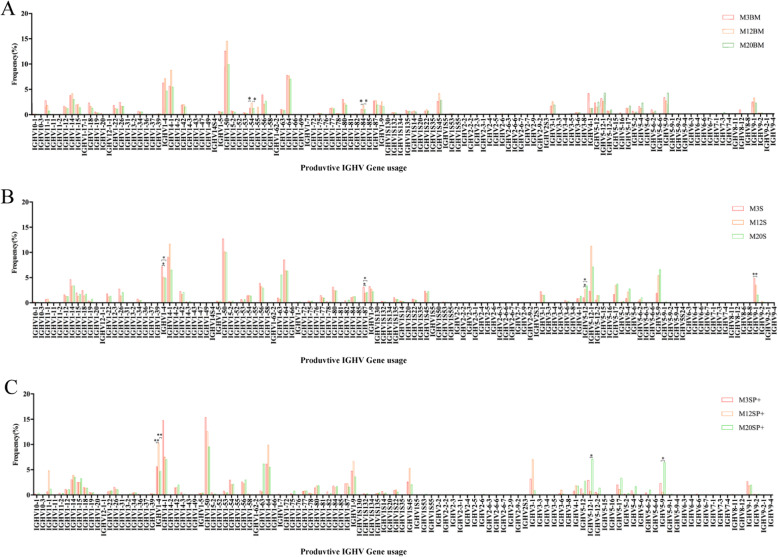
Productive sequence *IGHV* gene frequency in mice of different ages. **a** The gene frequency of the *IGHV* gene in the bone marrow B cells. **b** The gene frequency of the *IGHV* gene in the spleen B cells. **c** The gene frequency of the *IGHV* gene in the spleen memory B cells. The *p* values were determined using one-way ANOVA with a Bonferroni correction. All the statistically significant differences are indicated. * = *p* < 0.05, ** = *p* < 0.01, *** = *p* < 0.001

#### *D* gene usage (Fig. 3)

(1) Productive, pseudogene, and out-of-frame sequence high-frequency usage genes were concentrated in *IGHD1–1*, *IGHD2–1*, *IGHD2–3*, and *IGHD2–4*. (2) Productive sequences: Spleen B cell *IGHD2–5*, *IGHD3–3*, and *IGHD6–3* and spleen memory B cell *IGHD2–3*, *IGHD3–1*, IGHD3–2, and *IGHD3–3* showed significant differences with age (*p* < 0.05). (3) Pseudogene sequences: Bone marrow B cell *IGHD2–3*, and *IGHD2–4* and spleen B cell *IGHD3–1*, *IGHD3–3*, and *IGHD5–1* showed significant differences with changes in age (*p* < 0.05). (4) Out-of-frame sequences: The frequency of spleen B cell and memory B cell *IGHD2–4* in 20-month-old mice was greater than that in 12- and 3-month old mice; bone marrow B cell *IGHD2–2*, *IGHD2–4*, *IGHD3–2*, and *IGHD5–1*, spleen B cell *IGHD2–5*, and spleen memory B cell *IGHD1–3, IGHD2–1*, *IGHD2–12*, *IGHD2–5*, *IGHD3–1*, *IGHD3–2*, *IGHD4–1*, and *IGHD6–2* showed significant differences with changes in age (*p* < 0.05).

**Fig. 3:**
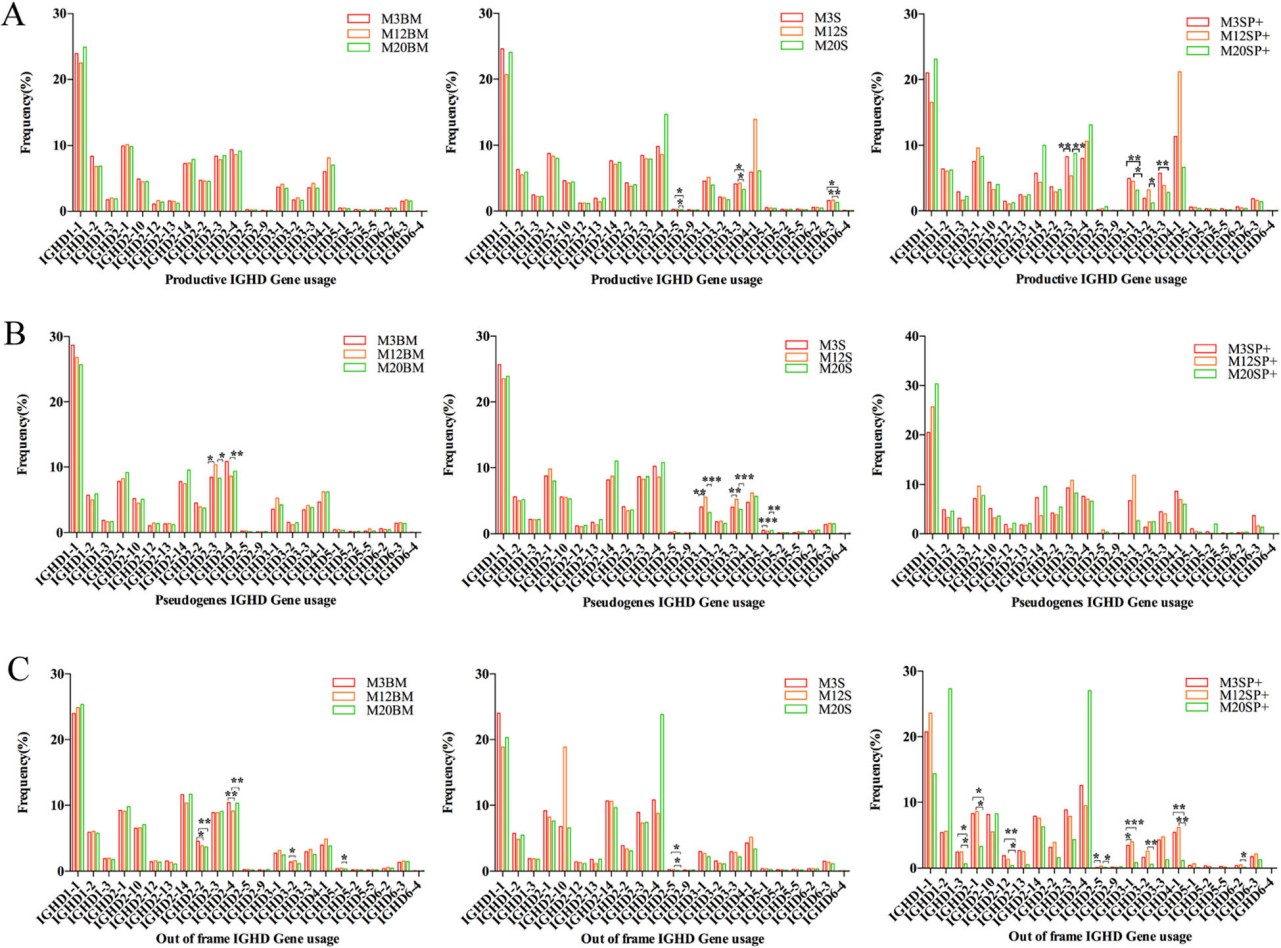
The *IGHD* gene frequency in the mice of different ages. **a** Productive sequence *IGHD* gene frequency. **b** Pseudogene sequence *IGHD* gene frequency. **c** Out-of-frame sequence *IGHD* gene frequency. The *p* values were determined using one-way ANOVA with a Bonferroni correction. All the statistically significant differences are indicated. * = *p* < 0.05, ** = *p* < 0.01, *** = *p* < 0.001

#### *J* gene usage (Fig. 4)

Productive sequences: The spleen B cell *IGHJ2* gene showed significant differences with changes in age (*p* < 0.05). Pseudogene sequences: The spleen B cell *IGHJ1* gene showed significant differences with changes in age (*p* < 0.05).

**Fig. 4:**
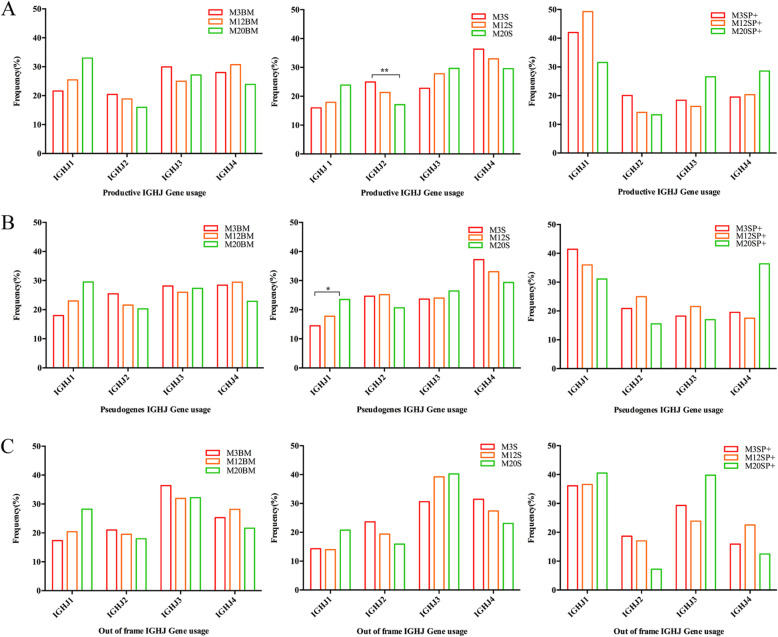
The *IGHJ* gene frequency in the mice of different ages. **a** Productive sequence *IGHJ* gene frequency. **b** Pseudogene sequence *IGHJ* gene frequency. **c** Out-of-frame sequence *IGHJ* gene frequency. The *p* values were determined using one-way ANOVA with a Bonferroni correction. All the statistically significant differences are indicated. * = *p* < 0.05, ** = *p* < 0.01

#### *V-J* pairing in the BCR H-CDR3 repertoire (Fig. S3–1, Fig. S3–2 and Fig. S3–3)

In the productive, pseudogene, and out-of-frame sequences, the *V-J* advantage pairing usage was performed differently. Cluster analysis showed that the bone marrow B cells, spleen B cells, and spleen memory B cells in the productive sequences had closer clustering distances at 12 months and 20 months. The pseudogene sequences had closer clustering of the bone marrow and spleen B cells at 3 months and 12 months, while spleen memory B cells were closer clustered at 12 months and 20 months. Out-of-frame sequences had closer clustering of bone marrow B cells and spleen memory B cells at 3 months and 12 months of age, while spleen B cells were closer clustered at 12 months and 20 months.

### Insertions and deletions of nucleotides in the BCR H-CDR3 repertoire

**Fig. 5:**
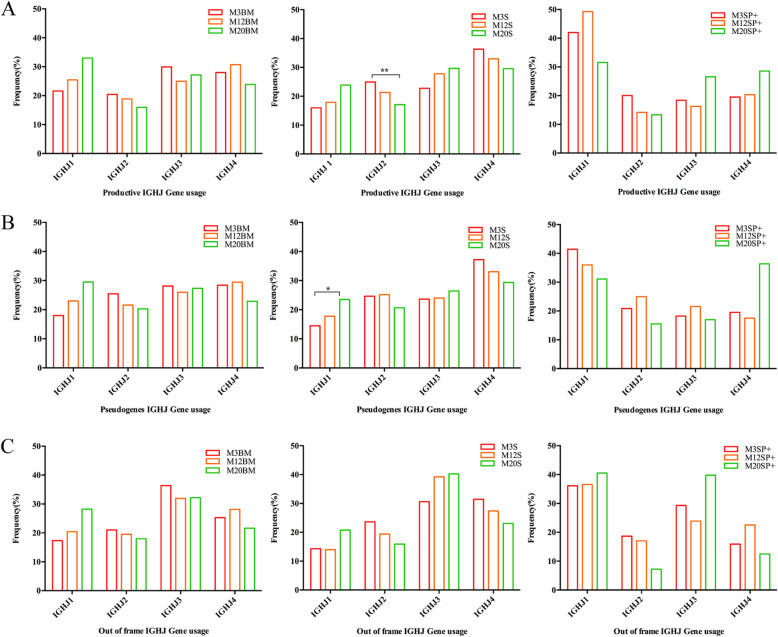
Insertion and deletion of nucleotides in the total sequences from mice of different ages. **a** Productive sequences. **b** Pseudogene sequences. **c** Out-of-frame sequences. The number of N1, N2, P3’V, P5’D and P5’J nucleotides added at the *V-D-J* junctions and the nucleotides deleted at 3’V, 5’D and 5’J by exonuclease trimming are shown (mean ± SD; error bars represent SD). The *p* values were determined using one-way ANOVA with a Bonferroni correction. All the statistically significant differences are indicated. * = *p* < 0.05, ** = *p* < 0.01, *** = *p* < 0.001

The CDR3 diversity results from the “N” nucleotides at the V → D(N1) and D → J(N2) junctions, exonuclease trimming (3′V trimmed, 5′D trimmed and 5′J trimmed) and the addition of palindromic “P” nucleotides (P3′V, P5′D and P5′J) [17]. According to the classification method of nucleotide insertion and deletion reported by Murugan et al. [18], it was found that in the productive, pseudogene, and out-of-frame sequences, bone marrow B cells had significant differences in 5’J trimming with age (*p* < 0.05); spleen B cell and memory B cell N1 insertion, N2 insertion, P5’D insertion, and 5’D trimming were significantly different (*p* < 0.05), (Fig. 5).

### Productive sequence clone proliferation in the BCR H-CDR3 repertoire

**Fig. 6:**
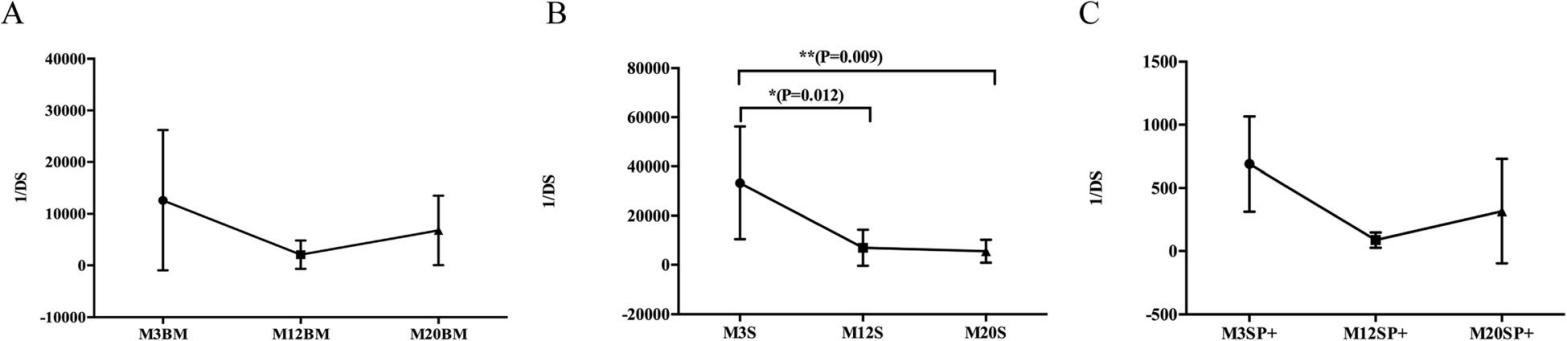
Diversity of the productive sequences of the BCR H-CDR3 repertoire in mice of different ages. **a** Bone marrow B cell repertoire diversity. **b** Spleen B cell repertoire diversity. **c** Spleen memory B cell repertoire diversity. 1/DS are shown (mean ± SD; error bars represent SD). The *p* values were determined using one-way ANOVA with a Bonferroni correction. All the statistically significant differences are indicated. * = *p* < 0.05, ** = *p* < 0.01

An analysis of productive sequence clone proliferation was conducted using the inverse of Simpson’s diversity index (1/DS), calculated as 1/DS = 1/∑{ni*(ni-1)}/{n*(n-1)}, where ni refers to the total number of the i-th sequence [19, 20]. The higher the 1/DS value, the richer the diversity and the lower the clonal proliferation [21]. The frequencies of the unique sequences are ordered from high to low, and a log10 scale is used on the Y-axis to reveal the frequency (Fig. S4). The BCR H-CDR3 repertoire of mice bone marrow B cells, spleen B cells and spleen memory B cells decreased with the increase in the 1/DS index (Fig. 6a). In the BCR H-CDR3 repertoire of mice spleen B cells, the 1/DS index at 12 months and 20 months was significantly lower than that at 3 months (*p* < 0.05) (Fig. 6b). The spleen memory B cell BCR H-CDR3 repertoire 1/DS index of 3-month-old mice was greater than that of 12- and 20-month-old mice (Fig. 6c).

### CDR3 length and amino acid usage in productive sequences

**Fig. 7:**
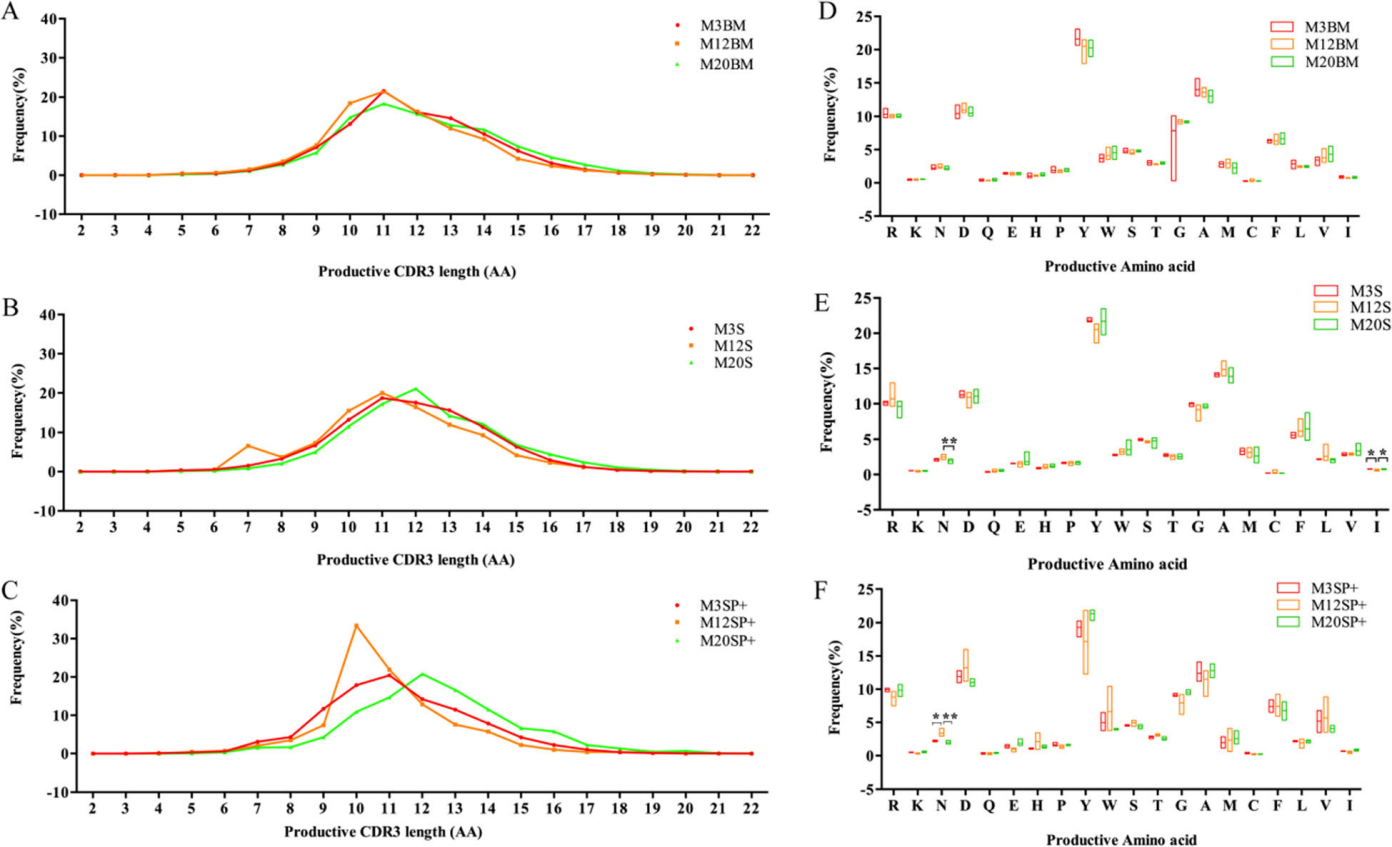
Productive sequence CDR3 length and AA usage for mice of different ages. **a** Bone marrow B cell CDR3 length. **b** Spleen B cell CDR3 length. **c** Spleen memory B cell CDR3 length. **d** Bone marrow B cell AA usage. **e** Spleen B cell AA usage. **f** Spleen memory B cell AA usage. All the statistically significant differences are indicated. * = *p* < 0.05, ** = *p* < 0.01

The distribution of CDR3 lengths at different ages and in different tissues was similar and showed a Gaussian distribution (Fig. 7a, b, c). There was a statistically significant difference in the use of asparagine (N) and isoleucine (I) in the spleen B cell repertoire with increasing age (*P* < 0.05). There was a statistically significant difference in the use of asparagine (N) in the spleen memory B cell repertoire with increasing age (*p* < 0.05), (Fig. 7d, e, f).

### Overlap in the productive sequences of the CDR3 repertoire

**Fig. 8:**
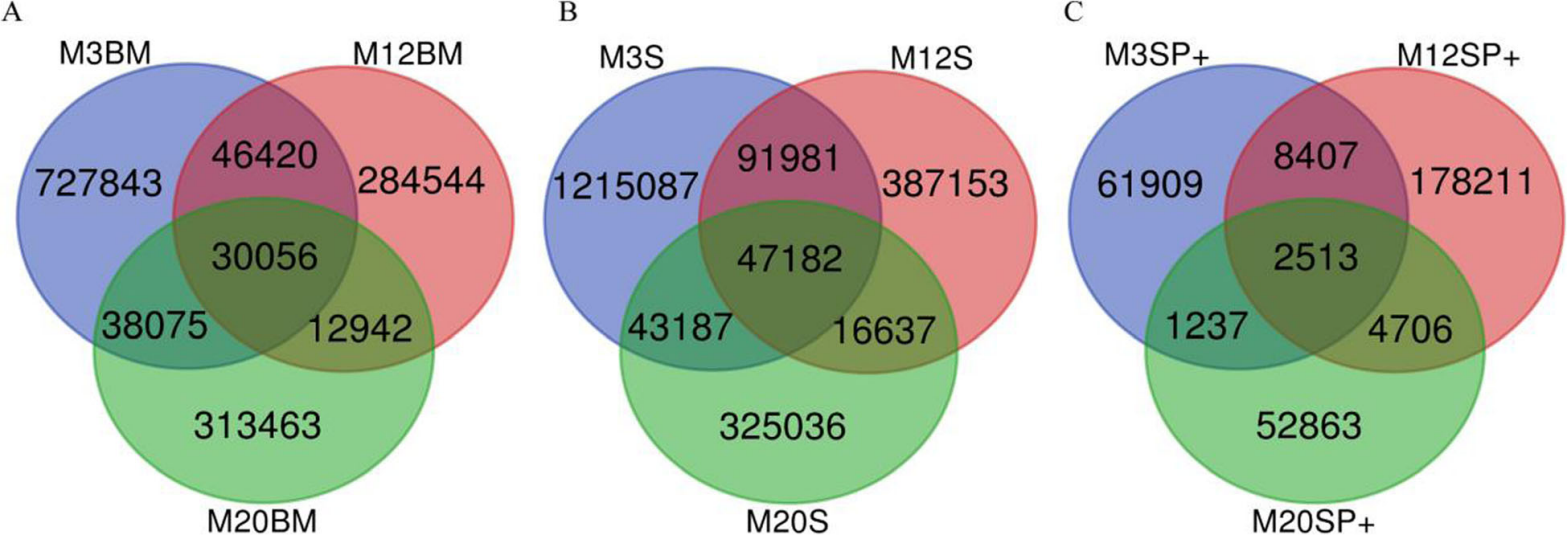
Overlap amino acid sequences of the BCR H-CDR3 repertoire in mice of different ages. **a** Overlap AA sequence of the CDR3 repertoire of bone marrow B cells. **b** Overlap AA sequence of the CDR3 repertoire of spleen B cells. **c** Overlap AA sequence of the CDR3 repertoire of spleen memory B cells

The ratio of overlap to the number of unique amino acids in the mice at different ages was calculated. The proportion of overlap in bone marrow and spleen B cells in 3-month old mice was lower than that in 12- and 20-month-old mice. This observation was not seen in spleen memory B cells (Fig. 8, Tables 2, 3 and 4).

**Table 2:** Statistical table of unique AA sequence overlap in the CDR3 sequences of bone marrow B cells in mice at different ages

Sample	M3BM Overlap (Overlap/Unique)	M12BM Overlap (Overlap/Unique)	M20BM Overlap (Overlap/Unique)
M3BM	1,016,234	76,476 (18.485%)	68,131 (15.685%)
M12BM	76,476 (7.525%)	413,723	42,998 (9.899%)
M20BM	68,131 (6.704%)	42,998 (10.393%)	434,374

**Table 3:** Statistical table of unique AA sequence overlap in the CDR3 sequences of spleen B cells in mice at different ages

Sample	M3S Overlap (Overlap/Unique)	M12S Overlap (Overlap/Unique)	M20S Overlap (Overlap/Unique)
M3S	1,750,816	139,163 (22.576%)	90,369 (18.834%)
M12S	139,163 (7.948%)	616,433	63,819 (13.301%)
M20S	90,369 (5.162%)	63,819 (10.353%)	479,821

**Table 4:** Statistical table of unique AA sequence overlap in the CDR3 sequences of spleen memory B cells in mice at different ages

Sample	M3SP+ Overlap (Overlap/Unique)	M12SP+ Overlap (Overlap/Unique)	M20SP+ Overlap (Overlap/Unique)
M3SP+	76,508	10,920 (5.069%)	3750 (6.007%)
M12SP+	10,920 (14.273%)	215,421	7219 (11.563%)
M20SP+	3750 (4.901%)	7219 (3.351%)	62,430

## Discussion

As the age of mice increases, the thymus and spleen T cells TCR CDR3 repertoire will change [22, 23], at the same time, the number and response capacity of central and peripheral B cells will change accordingly. The homogeneity and heterogeneity of the composition and characteristics of the central and peripheral B cells in younger/middle/old-aged mice is an important basis for the study of the responsive ability and mechanism of the aging immune system. In this experiment, 3-, 12-, and 20-month-old mice were used as subjects, and HTS was used to compare and analyze the homogeneity and heterogeneity of the productive, pseudogene, and out-of-frame sequences of the BCR H-CDR3 repertoire in bone marrow (central) and spleen (peripheral).

The study found that the white pulp of the spleen was more regular at 3 months of age, the density of lymphocytes was larger, and the shape of the shifting area is obvious. With the increase in the age of the mice, the structure of the white pulp became irregular at the ages of 12 months and 20 months. Some of them are relatively loose, with only a small number of lymphocytes, and the shape of the area became irregular. The white pulp structure is typically associated with antigenic stimulation; as the mice age increased, the mice received more antigenic stimulation, resulting in an irregular white pulp structure in aged mice, which is consistent with previous literature reports [24].

The diversity of the BCR H-CDR3 repertoire is derived from the *V(D) J* gene rearrangement, insertion, deletion, and high-frequency mutations in somatic cells. As mice age, changes in BCR H-CDR3 repertoire diversity can be demonstrated by random combinations of light and heavy chains [25]. The frequency of the *V*, *D*, and *J* genes was related to the advantages of naïve rearrangement, B cell self-tolerance selection, B cell clonal proliferation, immune response, etc. In theory, the frequency of the usage of the *V*, *D*, and *J* genes reflects the key features of the CDR3 recognition of specific antigens. This study found a high frequency of the usage of *IGHV1–4*, *IGHV14–1*, *IGHV1–50*, and *IGHV1–64* in the productive and out-of-frame sequences of bone marrow B cells, spleen B cells and spleen memory B cells at different ages. In the pseudogene sequences, the frequency of *IGHV1–67* and *IGHV1–83* was significantly higher than that of other genes; *IGHV1* had high-frequency usage at different ages and in different tissues, showing homogeneity, which is consistent with other researchers’ reports [8]. This finding suggests that in the naïve rearrangement, there is a significant advantage of some *V* gene in the naïve rearrangement, resulting in its high frequency in the self-tolerance selection and peripheral response BCR repertoire. In the productive sequences, mice bone marrow B cell *IGHV1–54* and *IGHV1–84;* spleen B cell *IGHV4–1*, *IGHV1–87*, *IGHV5–12*, and *IGHV9–1*; and spleen memory B cell *IGHV1–4*, *IGHV5–12–1*, and *IGHV5–9* usage significantly changed with age. Significant changes in gene usage may be related to B cell tolerance, clonal proliferation, and the frequency of the immune response and further indicate that the BCR H-CDR3 repertoire is partially heterogeneous at different ages and in different tissues. In the analysis of the frequency of *IGHV* gene family use, it was found that some *IGHV* gene families did not show regular changes with the increase of months (3 months, 12 months, and 20 months). This may require a more detailed analysis of the B cell subsets sorted in the bone marrow and spleen. The experiment needs to further clarify the characteristics of the naïve B cells and memory B cells (and different types of memory B and subsets) CDR3 repertoire in different parts of the bone marrow or spleen (Such as *IGHV* gene family utilization frequency) changes dynamically with the age of the month.

A pseudogene sequence is produced by the deletion of the initiation codon and the premature introduction of a termination codon [9]. A pseudogene sequence also participates in rearrangement. Because of the inefficiency of rearrangement, the pseudogene sequence better reflects the frequency characteristics of the *V*, *D* and *J* genes in the naïve rearrangement and together with the out-of-frame rearrangement of functional genes, it can be used to compare and analyze the composition and specificity of the productive rearrangement of functional genes [26].

The pseudogene sequence showed significant differences in bone marrow B cell *IGHV1–25*, *IGHV1–83, IGHD2–3* and *IGHD2–4* with age and significant differences in spleen B cell *IGHV1–32, IGHV1–67, IGHD3–1, IGHD3–3,* and *IGHD5–1* genes. Compared with the productive sequences, there were differences in *V* and *D* gene usage, suggesting that the pseudogene sequence better reflects naïve rearrangement. A out-of-frame sequence is the result of non-productive rearrangementwe. When we analyzed the *V* genes usage, we found that the out-of-frame sequence and the productive sequence have many similarities, mainly because the out-of-frame sequence is a translational reading frame change caused by insertion and deletion of nucleotides during gene rearrangement and cannot be translated into functionality AA. Therefore, we believe that the difference in the out-of-frame sequence in the bone marrow tissue with age changes is mainly affected by gene rearrangement, or it may be the cause of individual differences. Therefore, we believe that the difference in the age of the out-of-frame sequence in the central and peripheral tissues is mainly affected by gene rearrangement, or it may be affected by the antigen response in the periphery. The use of *V/D/J* families in B cells during mice aging is similar, which is consistent with the use of gene families during human aging [27]. There are some differences in the use of individual families, which is consistent with the study of the *V*, *D* and *J* genes in the peripheral blood of newborns and adults [28].

The closer the cluster analysis is, the more similar the gene access is. The cluster analysis of *V-J* pairing in spleen B cells and memory B cells shows that the clustering distances of 12 months and 20 months are the closest, while the cluster distance of 3 months is more. Indicating that the 12-month-old and 20-month-old genes are more similar, suggesting that it may be similar to the longer-term in vitro antigen stimulation, and B cells produce corresponding responses and clonal proliferation. The clustering distance of pseudogene sequences was closer at 3 months and 12 months than at 20 months in the bone marrow and spleen B cells. In the out-of-frame sequences in bone marrow B cells and spleen memory B cells at the ages of 3 months and 12 months, the clustering distance was closer than that at 20 months. It is suggested that more *V* and *J* gene naïve rearrangements have higher advantages and matching. The specific sources and mechanisms of various *V-J* pairing B cell populations need to explore its regular changes in experiments with more mice-month-old groups.

For the insertions and deletions of the BCR H-CDR repertoire, in the productive, pseudogene, and out-of-frame sequences, the bone marrow B cells had significant differences in 5’J trimming with age and may be mainly pro-B cells and pre-B cells. In the pro-B cell repertoire, the rearrangement is generally random, but there is a certain difference in the involvement of the *J* gene in functional and nonfunctional rearrangements. Therefore, in the next experiment, it is necessary to sort the pro-B cells and pre-B cells, naïve B cells and memory B cells in the bone marrow of mice of different months, and compare and analyze their characteristics in mice of different months. To explore the mechanism and significance of changes in central B cell rearrangement, selection and differentiation in the process of mice increasing with age. There were significant differences in spleen B cell and memory B cell N1 insertion, N2 insertion, P5’D insertion, and 5’D trimming, suggesting that the BCR H-CDR3 repertoire and B cell self-tolerance selection, immune response, and clonal proliferation are related. Due to the response of multiple autoantibodies and external antigens, the CDR3 repertoire participates in this process in addition to the naïve rearrangement, high-frequency mutations of somatic cells and the secondary rearrangement of the BCR, resulting in N1 insertion, N2 insertion, P5’D insertion, and 5’D trimming differences. Therefore, in the peripheral immune organs, it is more complicated to explore the mechanism and significance of insertion and deletion of the CDR3 repertoire of different subsets of B cells in the process of changing with the age of mice. It needs to be carried out on the basis of mice disease models of different months.

The diversity of the BCR H-CDR3 repertoire in different ages of mice showed that the diversity in bone marrow B cells and spleen memory B cells of 3-month-old mice was higher than that of 12- and 20-month old mice. In spleen B cells, the diversity of the 3-month-old mice repertoire was significantly higher than that of the 12- and 20-month old mice repertoire. This is consistent with previous studies, the diversity of the CDR3 repertoire in lymphoid tissues decreases with increasing months [22, 29]. The diversity of the BCR H-CDR3 repertoire is closely related to gene rearrangement and the immune response generated by external antigen stimulation. The diversity of the repertoire in the bone marrow is mainly due to the rearrangement/insertion/deletion of the *V*, *D*, and *J* genes [30]. As the mice age increases, the B cell output from the bone marrow to the periphery decreases, and the diversity decreases [5]. This is consistent with the decrease in the output of lymphocytes from the central to the periphery of the mice that the research group studied with the increase of months [22]. The findings may have occurred in the bone marrow, and this reduction may also reflect changes in the subsets [20, 25].

The response of spleen B cells to external antigen stimulation, as the body ages to increase the body’s depletion of B cells, also leads to a decrease in B cell diversity, and B cell clonal proliferation changes with increasing age. Not only are the unique CDR3 sequence species reduced, but the clonal proliferation that occurs is also greater. This phenomenon also indicates that the clonal distribution caused by the steady-state proliferation and peripheral selection in the aging process is more unbalanced. Related studies indicate that the imbalance of clonal distribution can reflect the response ability and the peripheral selection of self-identification [31]. The changes in the diversity of the central and peripheral BCR H-CDR3 repertoire in mice at 3, 12, and 20 months of age suggest that they are due to both central and peripheral causes, providing further insight into immune system aging and results from diversity studies, basic data and new research techniques.

The AA composition of the BCR H-CDR3 region was found to be high-frequency tyrosine in the B cells of different ages and tissue-derived B cells, consistent with the literature [32]. The use of isoleucine (I) in the spleen B cell repertoire was significantly higher at 12 months than at 3 months and 20 months, and the use of asparagine (N) was significantly lower at 20 months than at 12 months. In the spleen memory B cell repertoire, the frequency of asparagine (N) was significantly lower at 3 months and 20 months than at 12 months. It is suggested that the difference in AA in the peripheral BCR repertoire of mice at different ages may be related to the immune response generated by specific antigen stimulation.

The length distribution analysis of CDR3 revealed that the length of the bone marrow B cells, spleen B cells and spleen memory B cells of mice at different ages showed a Gaussian distribution with 11–12 AA residues. This is consistent with the result previously reported for mice CDR3, which was an average length of 11.5 ± 1.9 AA residues [17]. Compared with 3 months of age, the CDR3 length distribution in the BCR H-CDR3 repertoire of bone marrow B cells, spleen B cells, and spleen memory B cells shifted to the left at 12 months and shifted to the right at 20 months, suggesting that with the increase in age, recognition antigen-producing responses, mutations, and clonal proliferation have a tendency to become longer and may be related to the antigens that are exposed [20], which is consistent with the increase in the length of human CDR3 with age [33]. Pickman Y., et al. also found healthy elderly people’s BCR CDR3 length distributions can be distinguished from those of the young [34].

The overlap rate analysis of the B cell CDR3 repertoire is closely related to *VDJ* gene recombination selection, self-tolerance selection, and environmental immune response. This study found that the overlap ratio of the B cell CDR3 repertoire of mice of different months is higher (Fig. 8 and Tables 2, 3 and 4). The use of *IGHV*, *IGHD*, and *IGHJ* genes is roughly similar in different months of age, indicating that the extra shared CDR3s might result from the similar selection of *VDJ* genes that were more prone to result in the same amino acid sequences. The specific mechanism needs to be further explored, but through comparative analysis, it is found that the proportion of overlap in the bone marrow B cells and spleen B cells of mice at different ages was lower at 3 months and higher at 12 months and 20 months. The overlap of unique amino acids at different ages reflects not only clonal proliferation but also the presence of new, effective, unique sequences. Our results showed that the overlap rate of bone marrow and spleen B cells at 3 months was lower, and the overlap rate at 12 and 20 months was higher, which suggests that clonal proliferation increases with age, which is consistent with the previous reports in the literature [22]. Aranburu A., et al. found age-associated B cells (ABCs) in autoimmune mice are comprised of autoreactive MBCs expressing highly restricted H-CDR3 repertoires [35]. In our study, the spleen memory B cells have no obvious regularity, which is mainly affected by the stimulation of the external antigen response.

Although this study found that the CDR3 repertoire of bone marrow and spleen B cells and spleen memory B cells in mice have multiple heterogeneity and other characteristics with the change of months, However, it is necessary to further clarify the relationship between naïve B cells (CD19^+^IgD^+^CD27^−^) and memory B cells (CD45R^+^CD27^+^) in different parts of the bone marrow or spleen, and even the memory B cells (CD45R^+^CD27^+^) in the bone marrow and spleen need to be further sorting by the method, conduct research on the CDR3 repertoire, such as comparing the CDR3 repertoire of T-bet^−^ and T-bet^+^ memory B cells in the spleen germinal center or circulation with age [36]. Exploring the characteristics of B cell BCR in mice of different months of age can provide a basis for clinical disease mechanism research. For example, Zhang J., et al. found that B cell BCR activation is different in similar diseases of different ages, adult AML samples have significantly higher level of B cell activation and more secondary Ig class switch events than pediatric AML or non-tumor samples [37, 38].

## Conclusion

The degradation of the immune system related to aging is a dynamic process that affects the diversity and response capacity of the body’s immune repertoire. In this study, HTS was used to investigate the effects of aging on the characteristics of the BCR H-CDR3 repertoire in mice bone marrow B cells, spleen B cells and spleen memory B cells. Our data indicate that the diversity of the mice CDR3 repertoire decreases with increasing months. We found that the productive, pseudogene, and out-of-frame sequences of bone marrow B cells, spleen B cells and spleen memory B cells in 3-, 12-, and 20-month-old mice have different compositions, and some features show significant heterogeneity, which further provides a basis for investigating the decline and response of B cell immunity in younger/middle/older-aged mice.

## Methods

### Study subjects

Three-month-old, 12-month-old, and 20-month-old SPF female BALB/c mice were purchased from Chongqing Tengxin Biotechnology Co., Ltd., and introduced into the Central Laboratory Animal Center of Zunyi Medical University (SPF feeding conditions). All animals and experiments were performed in accordance with the guidelines of the Animal Care and Use of Laboratory Animals (Ministry of Health, China, 1998) and approved by the Laboratory Animal Ethics Committee of Zunyi Medical University.

### Sample preparation

(1) Mice bone marrow B cell samples (5 mice per month): 3 months old (M3BM): M3BM1, M3BM2, M3BM3, M3BM4, M3BM5; 12 months old (M12BM): M12BM1, M12BM2, M12BM3, M12BM4, M12BM5; and 20 months old (M20BM): M20BM1, M20BM2, M20BM3, M20BM4, M20BM5. (2) Mice spleen B cell samples (5 mice per month): 3 months old (M3S): M3S1, M3S2, M3S3, M3S4, M3S5; 12 months old (M12S): M12S1, M12S2, M12S3, M12S4, M12S5; and 20 months old (M20S): M20S1, M20S2, M20S3, M20S4, M20S5. (3) Mice spleen memory B cell samples (3 mice per month): 3 months old (M3SP^+^): M3S7, M3S8, M3S9; 12 months old (M12SP^+^): M12S7, M12S8, M12S9; and 20 months old (M20SP^+^): M20S7, M20S8, M20S9.

### Preparation of single-cell suspension

Mice were dissected to remove the spleen and bone marrow for tissue sectioning. A single-cell suspension of memory B cells (CD45R^+^CD27^+^) was prepared with Miltenyi bead sorting, and the purity of the sorted cells was identified. The medullary cavity was washed of the bone marrow cells with PBS to prepare a single-cell suspension, and then, the genomic DNA in the bone marrow and in the spleen single-cell suspension of the memory B cell sample was extracted.

### BCR H-CDR3 sequencing

This experiment is based on the gene composition of 16 large families of mice functional *IGHV*, pseudogene *IGHV* and ORF *IGHV*, and 4 families of mice *IGHJ*, we designed and synthesis of 16 upstream primers and 4 downstream primers of the mice, as well as 2 GAPDH primers, were conducted by Shanghai Invitrogen Biotechnology Co., Ltd. (Tab. S1–1; S1–2; S1–3).

The DNA was used as a template to PCR amplify the BCR H-CDR3 region, with 100–200 bp PCR product agarose gel recovery (Fig. S1), and the HTS of the CDR3 repertoire was completed by BGI.

### Data analysis

The CDR3 data of each mice at each month’s age were analyzed separately for composition characteristics. The CDR3 sequence of the HTS was converted into FASTA format and uploaded to the IMGT database (Use IMGT/HighV-QUEST tool and IMGT/GENE-DB database to provide total MUS data for analysis). The sequences with no results, unknown or < 90% V/J-region identity, AA junctions that did not have a C at the beginning or a W at the end, fewer than 6 nucleotides, blanks and ORFs were filtered out from the data downloaded by IMGT. The selected productive, pseudogene, and out-of-frame sequences were statistically analyzed using Excel, GraphPad Prism, HeIm, SPSS (one-way ANOVA), and Draw Venn Diagram online software. For statistical significance, * indicates *p* < 0.05, ** indicates *p* < 0.01, and *** indicates *p* < 0.001.

## Supplementary Information

**Additional file 1.** Supplemental Information can be found online at immunity.

## Data Availability

The datasets used and/or analyzed during the current study are available from the corresponding author on reasonable request.

## Abbreviations

1/DS: The inverse Simpson’s diversity index
AA: Amino acid
BCR: B Cell receptor
SPF: Specific pathogen free
H-CDR3: Heavy chain complementarity determining region 3
HTS: High-Throughput sequencing
IGHV: Immunoglobulin heavy chain variable gene
IGHD: Immunoglobulin heavy chain diversity gene
IGHJ: Immunoglobulin heavy chain jointing gene
ORF: Open reading frame
C: Cysteine
W: Tryptophan
