# Supplementary material for: Analysis of the heterogeneity of the BCR H-CDR3 repertoire in the bone marrow and spleen of 3-, 12-, and 20-month old mice
Source: Immun Ageing. 2021 Apr 12;18:17. doi: 10.1186/s12979-021-00231-2 (PMC8040230; doi:10.1186/s12979-021-00231-2)
Supplement: Supplementary file 1 — Additional file 1. Supplemental Information can be found online at immunity. [file 12979_2021_231_MOESM1_ESM.docx]

**Supplementary Figure**

**
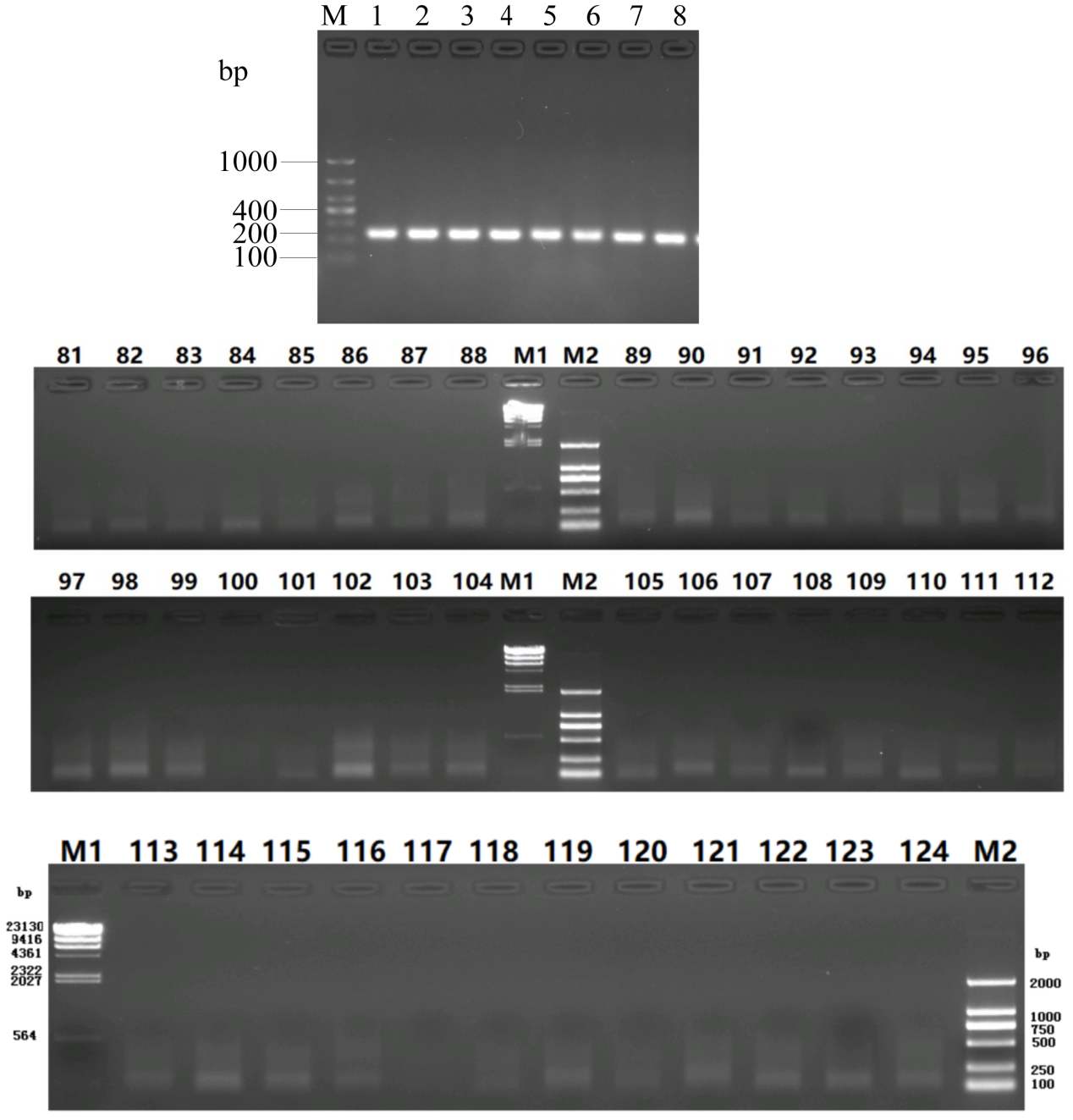
**

**Supplementary Figure 1.** Multiplex PCR product agarose gel recovery and identification electropherogram.

Note: The sample number corresponding to the serial number in the figure

| Serial number | Sample number | Serial number | Sample number | Serial number | Sample number | Serial number | Sample number |
| --- | --- | --- | --- | --- | --- | --- | --- |
| 1 | M3S1 | 87 | M20S3 | 98 | M20BM5 | 112 | M12BM2 |
| 2 | M3S2 | 88 | M20S4 | 101 | M12S1 | 113 | M12BM3 |
| 3 | M3S3 | 89 | M20S5 | 102 | M12S2 | 114 | M12BM4 |
| 4 | M3S4 | 90 | M20S7 | 103 | M12S3 | 115 | M12BM5 |
| 5 | M3S5 | 91 | M20S8 | 104 | M12S4 | 118 | M3S7 |
| 6 | M3BM1 | 92 | M20S9 | 105 | M12S5 | 119 | M3S8 |
| 7 | M3BM2 | 94 | M20BM1 | 107 | M12S7 | 120 | M3S9 |
| 8 | M3BM3 | 95 | M20BM2 | 108 | M12S8 | 122 | M3BM4 |
| 84 | M20S1 | 96 | M20BM3 | 109 | M12S9 | 123 | M3BM5 |
| 86 | M20S2 | 97 | M20BM4 | 111 | M12BM1 |  |  |


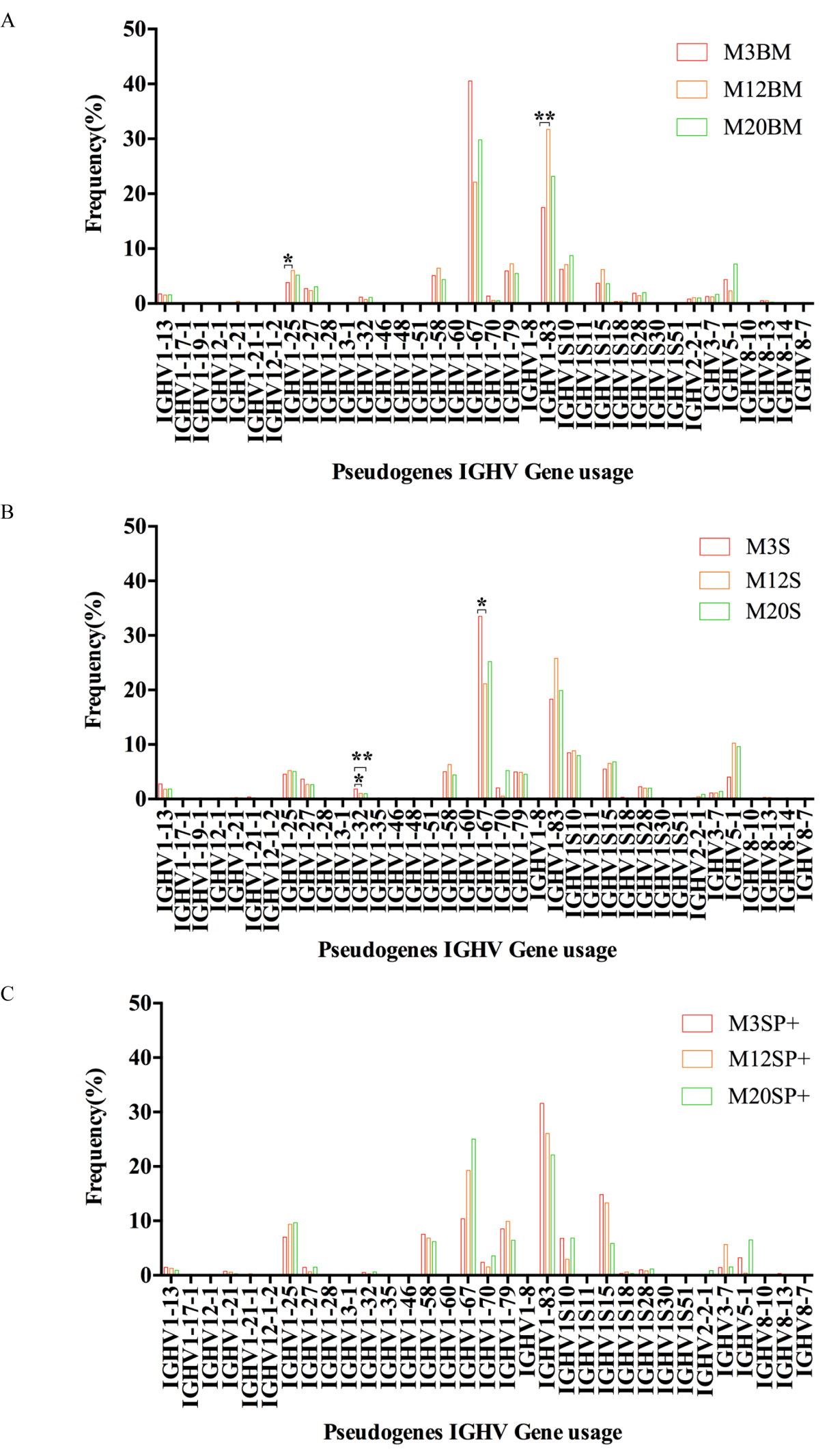


**Supplementary Figure 2-1.** Pseudogene sequence *IGHV* gene frequency in mice of different ages. **a** The gene frequency of the *IGHV* gene in the bone marrow B cells. **b** The gene frequency of the *IGHV* gene in the spleen B cells. **c** The gene frequency of the *IGHV* gene in the spleen memory B cells. The p values were determined using one-way ANOVA with a Bonferroni correction. All the statistically significant differences are indicated. * = *p* < 0.05, ** = *p* < 0.01.


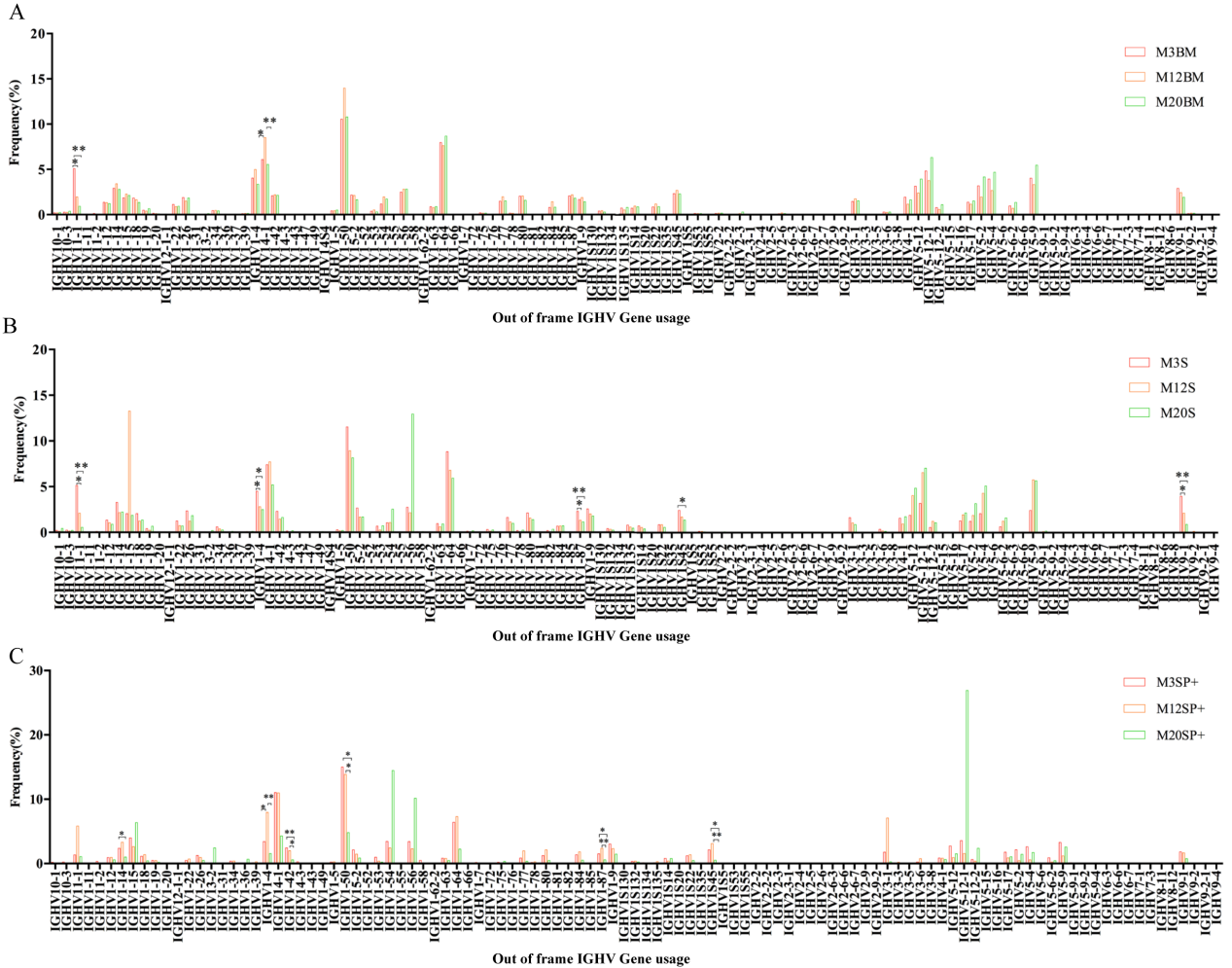


**Supplementary Figure 2-2.** Out-of-frame sequence *IGHV* gene frequency in mice of different ages. **a** The gene frequency of the *IGHV* gene in the bone marrow B cells. **b** The gene frequency of the *IGHV* gene in the spleen B cells. **c** The gene frequency of the *IGHV* gene in the spleen memory B cells. The p values were determined using one-way ANOVA with a Bonferroni correction. All the statistically significant differences are indicated. * = *p* < 0.05, ** = *p* < 0.01.


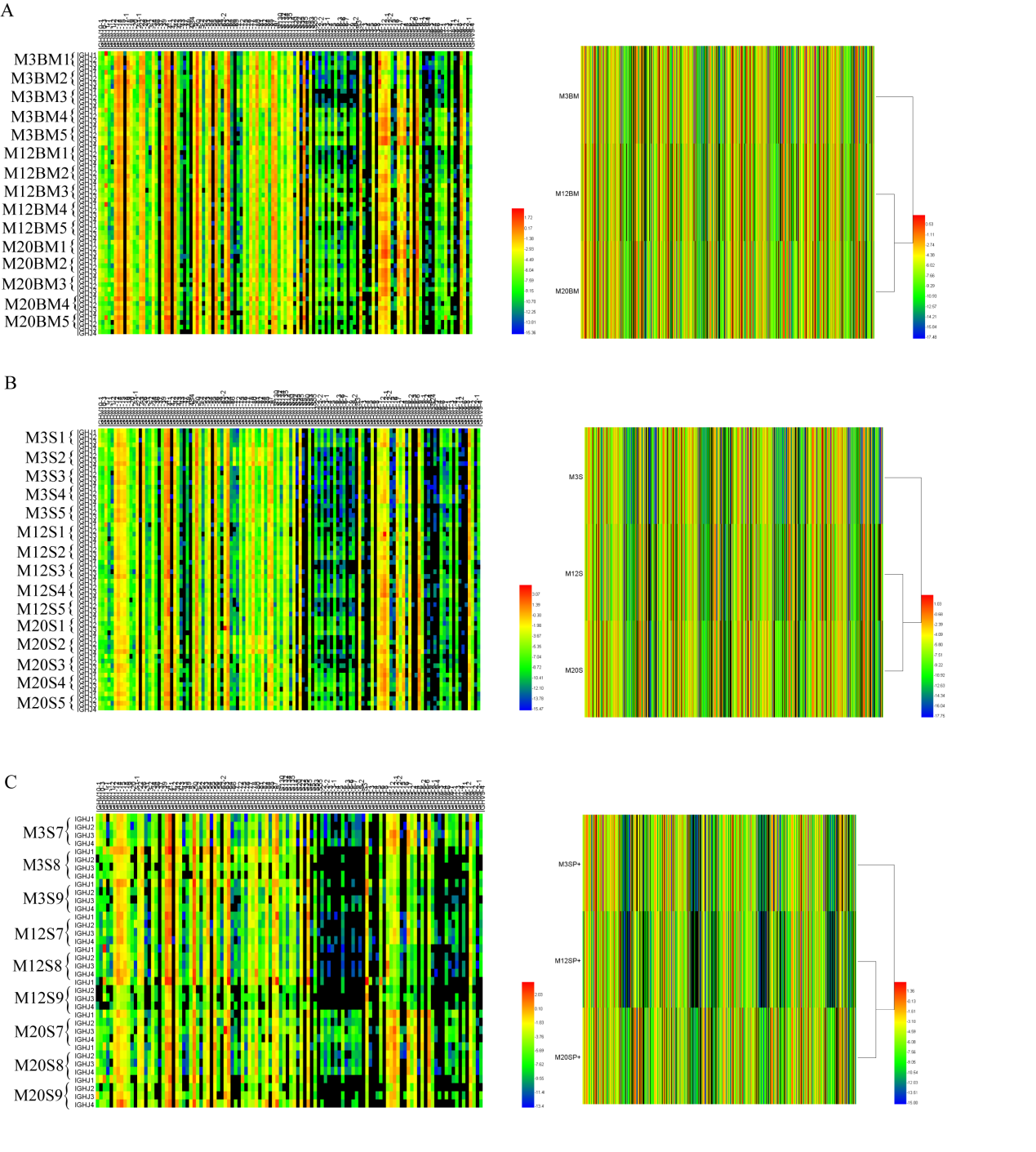


**Supplementary Figure 3-1.** Pairing and cluster analysis of the *V-J* gene in the productive sequences of mice of different ages. **A.** Bone marrow B cell productive sequence *V-J* gene pairing (left) and cluster analysis (right). **B.**  Spleen B cell productive sequence *V-J* gene pairing (left) and cluster analysis (right). **C.** Spleen memory B cell productive sequence *V-J* gene pairing (left) and cluster analysis (right).


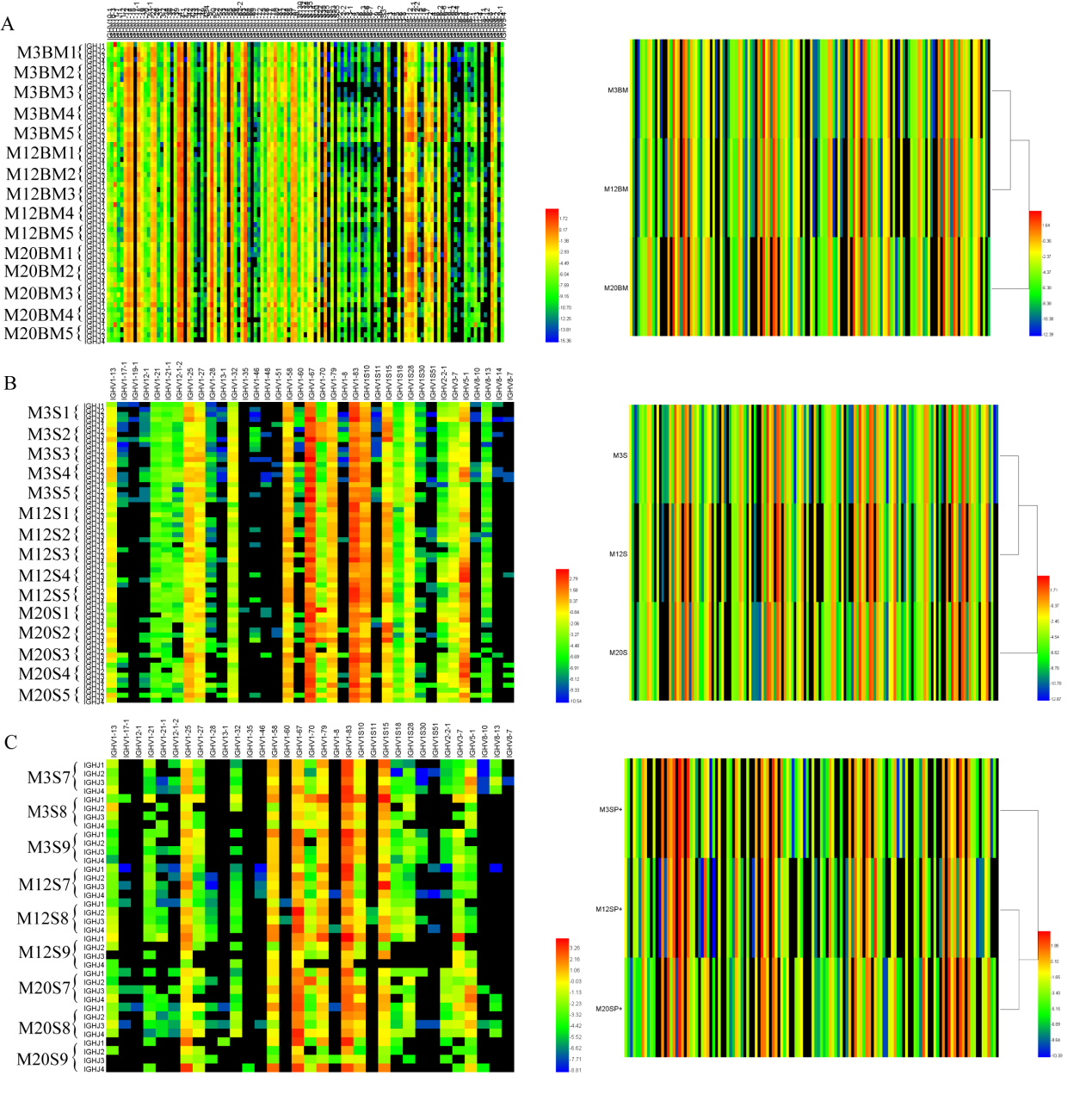


**Supplementary Figure 3-2.** Pairing and cluster analysis of the *V-J* gene in the pseudogene sequences of mice of different ages. A. Bone marrow B cell productive sequence *V-J* gene pairing (left) and cluster analysis (right). B. Spleen B cell productive sequence *V-J* gene pairing (left) and cluster analysis (right). C. Spleen memory B cell productive sequence *V-J* gene pairing (left) and cluster analysis (right).


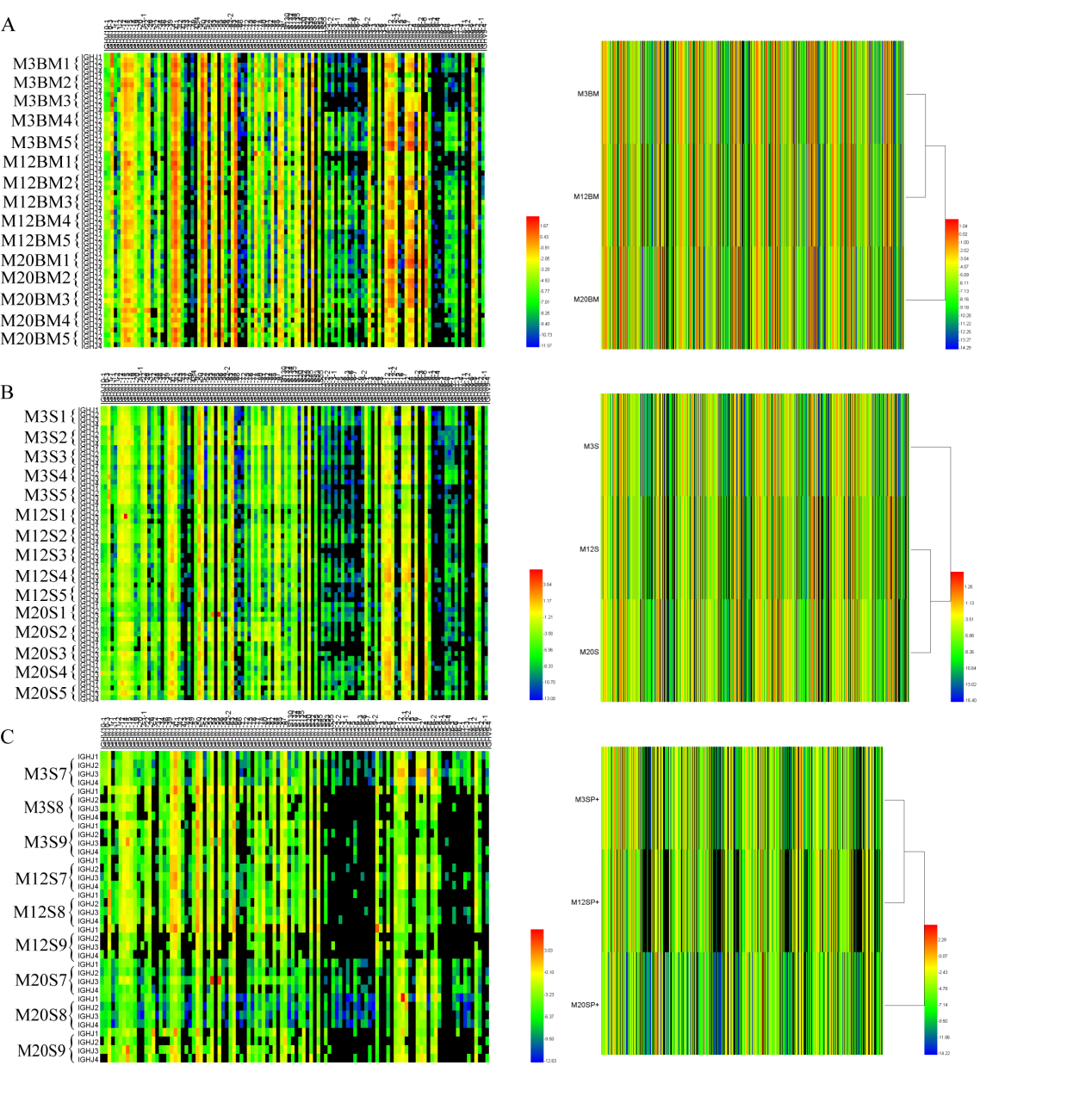


**Supplementary Figure 3-3.** Pairing and cluster analysis of the *V-J* gene in the out-of-frame sequences of mice of different ages. A. Bone marrow B cell productive sequence *V-J* gene pairing (left) and cluster analysis (right). B. Spleen B cell productive sequence *V-J* gene pairing (left) and cluster analysis (right). C.Spleen memory B cell productive sequence *V-J* gene pairing (left) and cluster analysis (right).


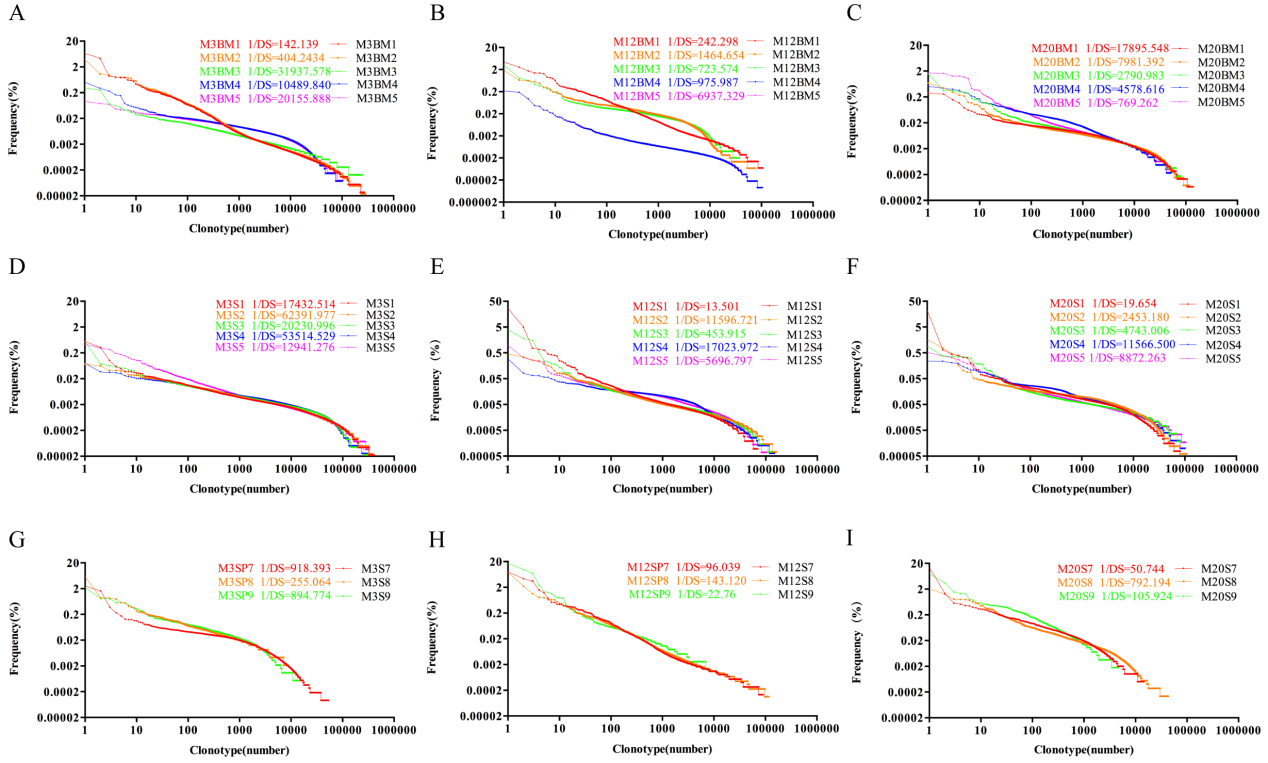


**Supplementary Figure 4.** Clonal distribution of the BCR H-CDR3 repertoire in mice of different ages. A. Clonal distribution of bone marrow B cell repertoire in mice at 3 months of age. B. Clonal distribution of bone marrow B cell repertoire in mice at 12 months of age. C. Clonal distribution of bone marrow B cell repertoire in mice at 20 months of age. D. Clonal distribution of spleen B cell repertoire in mice at 3 months of age. E. Clonal distribution of spleen B cell repertoire in mice at 12 months of age. F. Clonal distribution of spleen B cell repertoire in mice at 20 months of age. G. Clonal distribution of spleen memory B cell repertoire in mice at 3 months of age. H. Clonal distribution of spleen memory B cell repertoire in mice at 12 months of age. I. Clonal distribution of spleen memory B cell repertoire in mice at 20 months of age.

**Supplementary Table S1-1.** The upstream *IGHV* primer (5'-3') for mice BCR H-CDR3 repertoire

| **Name of Primer** | **IGHV Gene Sequence(5^’^-3^’^)** |
| --- | --- |
| *IGHV1* | CCTCCAGCACAGCCTACTG |
| *IGHV2* | TCCAAGAGCCAAGTTTTCTT |
| *IGHV3* | GTTGAATTCTGTGACTACTGAGG |
| *IGHV4* | GCCAAAAATACGCTGTACCTG |
| *IGHV5* | CGATTCACCATCTCCAGAGAC |
| *IGHV6* | GATGATTCCAAAAGTAGTGTC |
| *IGHV7* | TTCCCAAAGCATCCTCTATC |
| *IGHV8* | ATACCTCCAACAACCAGGTA |
| *IGHV9* | TCTGCCAGCACTGCCTATTT |
| *IGHV10* | CCAGAGATGATTCACAAAGC |
| *IGHV11* | CCTGCAGATGAGCAATGTGC |
| *IGHV12* | TTTATCCAGCTGAGCTCTGT |
| *IGHV13* | ATGCAGAGTCTGTGAAAGGC |
| *IGHV14* | CATCCTCCAACACAGCCTAC |
| *IGHV15* | TGTCCAACACAGCCTACTTG |
| *IGHV16* | GACAGATTCTCCATCTCCAG |

**Supplementary Table S1-2.** The downstream *IGHJ* primer (5'-3') for mice BCR H-CDR3 repertoire

| **Name of Primer** | **IGHC Gene Sequence(5^’^-3^’^)** |
| --- | --- |
| *IGHJ1* | TGAGGAGACGGTGACCGTGGTC |
| *IGHJ2* | AGGAGACTGTGAGAGTGGTGCC |
| *IGHJ3* | CAGAGACAGTGACCAGAGTCCC |
| *IGHJ4* | CGGTGACTGAGGTTCCTTGACC |

**Supplementary Table S1-3.** The GAPDH sequence of the upstream and downstream primers (5'-3') of mice

| **Name of Primer** | **IGHC Gene Sequence(5^’^-3^’^)** |
| --- | --- |
| GAPDH sense | GGTGAAGGTCGGTGTGAACG |
| GAPDH anti-sense | CTCGCTCCTGGAAGATGGTG |
